# Electrochemical Evaluation of* trans*-Resveratrol Levels in Red Wine Based on the Interaction between Resveratrol and Graphene

**DOI:** 10.1155/2017/5749025

**Published:** 2017-07-27

**Authors:** Lantao Liu, Yanli Zhou, Yiyu Kang, Haihong Huang, Congming Li, Maotian Xu, Baoxian Ye

**Affiliations:** ^1^Henan Engineering Laboratory of Green Synthesis for Pharmaceuticals, College of Chemistry and Chemical Engineering, Shangqiu Normal University, Shangqiu 476000, China; ^2^Henan Key Laboratory of Biomolecular Recognition and Sensing, College of Chemistry and Chemical Engineering, Shangqiu Normal University, Shangqiu 476000, China; ^3^College of Chemistry and Molecular Engineering, Zhengzhou University, Zhengzhou 450001, China

## Abstract

*trans*-Resveratrol is often considered as one of the quality standards of red wine, and the development of a sensitive and reliable method for monitoring the* trans*-resveratrol levels in red wine is an urgent requirement for the quality control. Here, a novel voltammetric approach was described for probing* trans*-resveratrol using a graphene-modified glassy carbon (GC) electrode. The proposed electrode was prepared by one-step electrodeposition of reduced graphene oxide (ERGO) at a GC electrode. Compared with the bare GC electrode, the introduced graphene film on the electrode surface dramatically improved the sensitivity of the sensor response due to the *π*-*π* interaction between the graphene and* trans*-resveratrol. The developed sensor exhibited low detection limit of 0.2 *μ*M with wide linear range of 0.8–32 *μ*M and high stability. For the analysis of* trans*-resveratrol in red wine, the high anti-interference ability and the good recoveries indicated the great potential for practical applications.

## 1. Introduction

Resveratrol (3,5,4′-trihydroxystilbene), a member of the stilbene family of phenolic compounds, has been reported to have health benefits because it could provide antioxidative and carcinogenic protection, reduce platelet aggregation, and then decrease the incidence of coronary heart disease [1]. Resveratrol is present in grapes, peanuts, and mulberries, and it is noted that a large amount of phenolic compounds in skins, seeds, and pulp of grapes is partially extracted during winemaking [2]. Red wine seems to be an essential component and moderate drinking could be responsible for health-promoting properties. Phenolic compounds are related to several sensorial characteristics such as color, flavor, astringency, and hardness of red wine, and particularly* trans*-resveratrol has become one of the quality standards of red wine [3, 4]. Therefore, the rapid, sensitive, and accurate determination of* trans*-resveratrol in red wine is very important for the improvement of manufacturing process, the control of storage condition, and the evaluation of nutrition value.

Due to the important physiological role and the significance of* trans*-resveratrol level, several analytical methods including liquid chromatography [5–11], capillary electrophoresis [12–15], fluorescent assays [16, 17], spectroscopy technique [18, 19], and molecularly imprinted polymer [20, 21] have been reported for the analysis of* trans*-resveratrol. The limitation of the fluorescent assays and spectroscopy technique is the substance interference from the real samples [22]. The* trans*-resveratrol detection by chromatographic, electrophoretic, and molecularly imprinted polymer has high sensitivity and specificity, but these methods are time-consuming and/or need expensive instruments. Electrochemical methods can allow for accurate and direct determination of potentials of redox-active molecules. However, electrochemical detection of* trans*-resveratrol using bare electrodes is difficult owing to its high overpotential and the interference of coexisting phenolic compounds in red wines. The electrochemical sensors have been designed for the* trans*-resveratrol detection, while some limitations including complicated fabrication process or low sensitivity still exist [23–31]. Hence, the search for reliable material for the modification on the electrode surface to determine* trans*-resveratrol is of considerable interest.

Among the modified materials, graphene has attracted tremendous attentions as an ideal material for electrochemistry because of its large surface area, large 2D electrical conductivity, and low cost [32]. So far, most of the graphene immobilized on electrode surface by drop-casting was synthesized by chemical reduction of graphene oxide sheets. Toxic reducing agents were needed in the chemical methods, and the preparation of the graphene-modified electrodes involved a coating process to keep their stability [33]. Recently, the modification of graphene on electrode surfaces by electrochemical methods has been certified to be a promising strategy due to its fast, simple, stable, and green nature [34]. Our group reported that a graphene film on the electrode surface was formed from graphene oxide dispersion by one-step electrodeposition, and the introduced graphene layer facilitates the electron transfer between the analyte and the electrode surface [35, 36]. In addition, resveratrol has strong interaction with the graphene-related materials by their *π*-*π* stacking [37]. In this regard, the graphene layer is expected to accumulate* trans*-resveratrol on the electrode surface and promote its electron transfer.

Herein, a new electrochemical sensing platform for* trans*-resveratrol detection was developed based on graphene-modified electrode prepared by one-step electrodeposition of graphene oxide. The electrochemical behavior of the* trans*-resveratrol was studied on the graphene-modified electrode. The analytical characteristics including sensitivity, detection range, and stability of this sensing system were investigated. The further determination for red wine samples was also researched to test the effectivity of the method for food quality control. This strategy for* trans*-resveratrol detection is advantageous in terms of its simplicity, sensitivity, high stability, and anti-interference ability.

## 2. Materials and Methods

### 2.1. Chemical and Reagents

Graphene oxide was obtained from graphite nanopowders (Sinopharm Chemical Reagent Co., China) by a modified Hummers method [35].* trans*-Resveratrol was purchased from Sigma-Aldrich. 0.04 M Britton-Robinson (BR) buffer solution was prepared from an acidic solution which contained 0.04 M H_3_PO_4_, HOAc, and H_3_BO_3_ by adjusting to different pH using 0.2 M NaOH. All other reagents were supplied by commercial suppliers and used without a filtration system. The water used was purified by a Millipore M-Q purification system (>18 MΩ).

### 2.2. Preparation of the Graphene-Modified Electrode

Glassy carbon (GC, 3 mm in diameter, Shanghai CH Instruments, China) surfaces were polished with 1.0, 0.3, and 0.05 *μ*m alumina powder using pure water on polishing cloths until a mirror finish was obtained. The electrodes were sonicated for 5 min to remove the alumina residues followed by rinsing with pure water and acetone. After polishing, 15 consecutive cycles from 0.5 to −1.5 V (versus Ag/AgCl) were carried out on the GC electrodes in a 0.5 g L^−1^ graphene oxide dispersion solution containing 0.1 M KCl at a scan rate of 50 mV s^−1^ [35, 36]. Thus, the electrodeposition of reduced graphene oxide-modified GC (ERGO/GC) electrodes was fabricated, and the working electrode was stored at 4°C in refrigerator prior to use. The surface morphologies of the modified electrodes were examined with scanning electron microscopy (SEM; Quonxe-2000, FEI).

### 2.3. Electrochemical Measurements

All electrochemical measurements were carried out on a CHI 660D electrochemical workstation (Shanghai CH Instruments, China). A conventional three-electrode system was used with a modified GC disk electrode, a platinum foil, and a saturated Ag/AgCl as working electrode, counter electrode, and reference electrode, respectively. The voltammetric responses of* trans*-resveratrol on the ERGO/GC electrodes were recorded by differential pulse voltammetry (DPV) from 0 to −0.5 V with a pulse amplitude of 0.005 V and a pulse width of 0.1 s. Prior to each electrochemical measurement, the buffer solutions were purged with high-purity N_2_ gas for at least 15 min. All electrochemical measurements were performed at room temperature.

### 2.4. Determination of* trans*-Resveratrol in Red Wine

The red wine samples (Greatwall, China) were purchased from local supermarkets. At first, 6.0 mL of sodium hydrogen tartrate/disodium tartrate buffer solution (0.26 M) was added to 0.50 mL of red wine in a separatory funnel. The mixed solution was extracted with 5.0 mL of diethyl ether by shaking vigorously for 60 s, and then the upper organic phase was separated and evaporated to dryness by a stream of N_2_ gas at room temperature. Finally, the residue was diluted to 10.0 mL using 0.04 M BR (pH 2.0) solution, and the DPV curves were recorded for the detection of* trans*-resveratrol in the red wine.

## 3. Results and Discussion

### 3.1. Characterization of the ERGO/GC Electrode

**Scheme 1:**
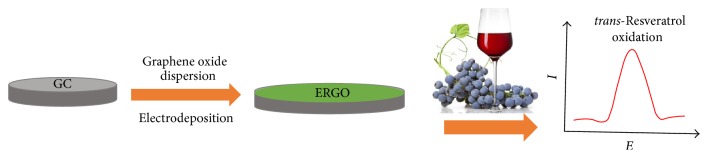
Illustrations of the preparation of the ERGO/GC electrode and the detection of* trans*-resveratrol.

**Figure 1:**
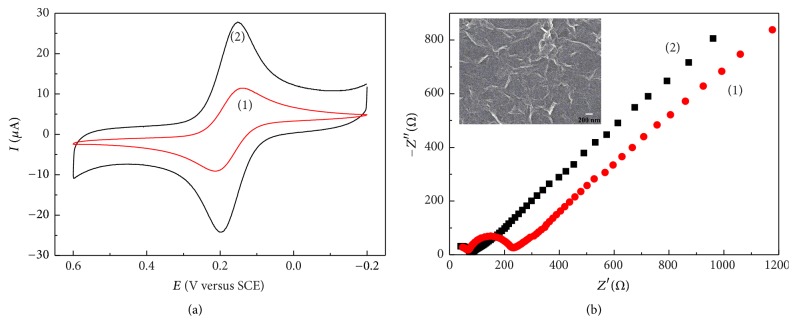
(a) CV curves of 1 mM [Fe(CN)_6_]^3−/4−^ in 0.1 M PBS (pH 7.0) at a scan rate of 50 mV s^−1^ and (b) Nyquist plots of 10 mM [Fe(CN)_6_]^3−/4−^ in 0.1 M KCl from 0.1 MHz to 0.1 Hz at ac amplitude of 5 mV under open-circuit potential conditions, obtained at the GC (1) and ERGO/GC (2) electrodes. Inset of (b) is the SEM image for the ERGO/GC electrode.

Illustrations of the preparation of the ERGO/GC electrode and the detection for* trans*-resveratrol were shown in Scheme 1. The formation of ERGO film on the electrode surface was directly verified by SEM characterization (Figure 1(b)). The presence of thin, aggregated, and crumpled graphene sheets associated with a wrinkled texture was observed, indicating their high surface area. To compare the charge transfer property of the GC electrode before and after the modification of ERGO, cyclic voltammetry (CV) for 1 mM [Fe(CN)_6_]^3−/4−^ in 0.1 M PBS (pH 7.0) was performed at the GC and ERGO/GC electrodes, as shown in Figure 1(a). Well-defined CV curves of [Fe(CN)_6_]^3−/4−^ on the two electrodes were obtained, indicating nearly reversible or quasi-reversible electron-transfer kinetics for both interfaces. However, compared with GC electrode, there was an obvious increase in the cathodic and anodic current responses and a significant decrease in the potential difference (Δ*E*_*p*_ from 73 to 48 mV) at the ERGO/GC electrode. The results might be attributed to the plenty of surface area and the high electric conductivity of ERGO film presented on the surface of GC electrode.

The ERGO/GC electrode was further evaluated by electrochemical impedance spectroscopy (EIS) in 0.1 M KCl containing 5 mM [Fe(CN)_6_]^3−/4−^, as illustrated in Figure 1(b). The semicircle portion appearing at high frequencies corresponds to the charge transfer limiting process, and the electron-transfer resistance (*R*_et_) could be directly measured from the semicircle diameter. At the bare GC electrode, the *R*_et_ value could be estimated to be 76 ± 3 Ω. After the modification of ERGO on the GC electrode, the *R*_et_ values decreased nearly to zero, revealing that the addition of graphene film formed high electron conduction pathways between the electrode surface and probe owing to less oxygen on the ERGO surface. Moreover, in comparison with the chemical reduction of graphene oxide modified on the electrode surface by drop-casting, the prepared ERGO sheets possess some advantages such as (1) simplicity by one-step electrochemical deposition, (2) green nature without toxic reducing agents, (3) controllable film thickness by the electrochemical cycle number, and (4) high stability due to its poor insolubility. Therefore, the ERGO film is an ideal substrate to study the redox properties of electroactive molecules.

### 3.2. Voltammetric Responses of* trans*-Resveratrol on the ERGO/GC Electrode

**Figure 2:**
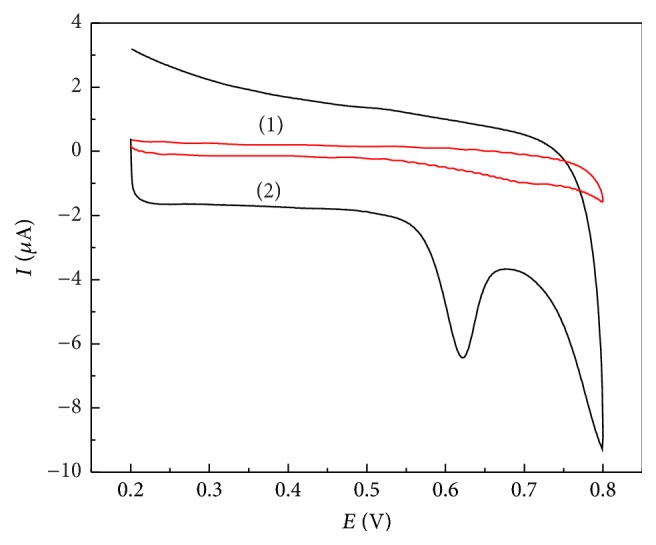
CV curves of 20 *μ*M* trans*-resveratrol in 0.04 M BR (pH 2.0) solution at the GC (1) and ERGO/GC (2) electrodes. Scan rate: 25 mV s^−1^.

Figure 2 shows a comparison of CVs for* trans*-resveratrol obtained at the bare GC and ERGO/GC electrodes. As expected, at the ERGO/GC electrode, an oxidation peak located at 0.62 V for* trans*-resveratrol was remarkably observed. At the bare GC electrode, only a low background current was observed. The above results are attributed to the high surface area of ERGO film and the rapid electron transfer between* trans*-resveratrol and the modified electrode surface. Furthermore, the hydrogen-bond interactions and/or strong aromatic *π*-*π* stacking between the resveratrol and graphene make the* trans*-resveratrol enrichment on the electrode surface. Thus, the ERGO film introduced on the electrode surface is indeed a useful addition for* trans*-resveratrol detection.

### 3.3. Mechanistic Studies of* trans*-Resveratrol Oxidation on the ERGO/GC Electrode

**Figure 3:**
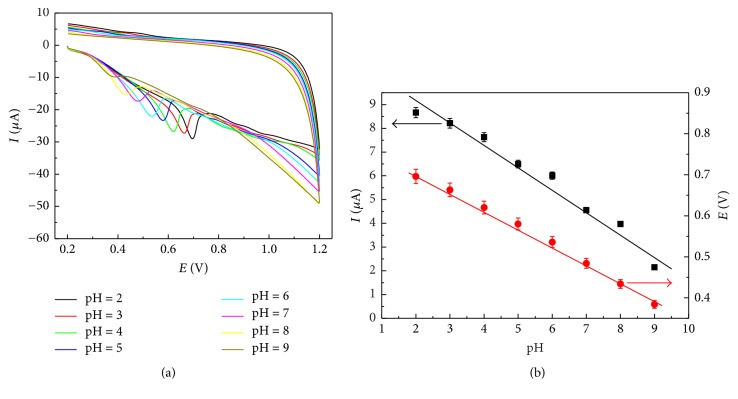
(a) CV curves of 20 *μ*M* trans*-resveratrol in 0.04 M BR solution with different pH at the ERGO/GC electrode. Scan rate: 25 mV s^−1^. (b) Effect of pH value on oxidation peak current and peak potential.

**Figure 4:**
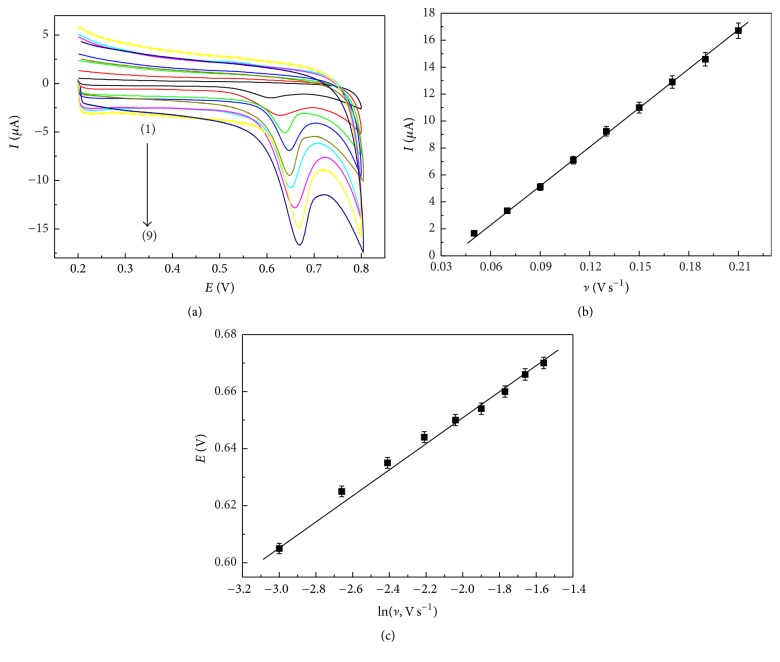
(a) CV curves of 20 *μ*M* trans*-resveratrol in 0.04 M BR solution (pH 2.0) at the ERGO/GC electrode with different scan rate: (1) 50, (2) 70, (3) 90, (4) 110, (5) 130, (6) 150, (7) 170, (8) 190, and (9) 210 mV s^−1^. Effect of scan rate on oxidation peak current (b) and peak potential (c).

Figures 3 and 4 illustrate the effects of the pH values and scan rates in the 0.04 M BR buffer solution for the* trans*-resveratrol responses at the ERGO/GC electrode, respectively. The potential of the oxidation peak shifted to more negative potentials between pH 2.0 and 9.0 in Figure 3, demonstrating the participation of the proton transfer on the electrode surface. The peak current decreased with the increasing of pH value from 2.0 to 10.0, which could be attributed to a difference in adsorption of its acidic and basic form. In consideration of buffer range, the 0.04 M BR buffer solution (pH 2.0) was selected as the supporting electrolyte for the following studies. In Figure 4(a), the peak currents of* trans*-resveratrol increased with the increase of the scan rates. The peak current of* trans*-resveratrol exhibited linear relationship with scan rate in the range of 0.05–0.21 V s^−1^ (Figure 4(b)). The linear regression equation was *I*_*p*_ = −3.21 + 94.47*υ* (*R* = 0.999), suggesting that the absorption-controlled behavior predominated in this process.

In Figure 4(c), the oxidation peak potential shifted to less positive values with the increase of scan rates, and a linear correlation between the peak potential and the natural logarithm of scan rate was *E*_*p*_ = 0.74 + 0.043 ln *υ* (*R* = 0.997). The relationship between the oxidation peak potential and the scan rate for the irreversible electrode process obeys the following equation:

(1) $$\begin{array}{c} E_{p}=E^{0\prime }+\left(\;\frac{RT}{\alpha n_{a}F}\;\right)\ln\left(\;\frac{RTk^{0}}{\alpha n_{a}F}\;\right)+\left(\;\frac{RT}{\alpha n_{a}F}\;\right)\ln\nu , \end{array}$$

where *E*^0′^ is the formal potential, *α* is the transfer coefficient, *n*_*a*_ is the number of the electrons transferred in the rate-determining step, *k*^0^ is the electrochemical rate constant, *R* is the gas constant, *T* is the temperature, and *F* is the Faraday constant. The value of *αn*_*a*_ was calculated to be 0.59 from the slope (0.043) of the linear correlation. The value of*α* was from 0.3 to 0.7 for most systems. Thus, assuming that *α* = 0.5, *n*_*a*_ was estimated to be 1 for the oxidation of* trans*-resveratrol at the ERGO/GC electrode. In addition, the relationship of the oxidation peak potential and pH value in Figure 3(b) was in accord with the following equation: *E*_*p*_(*V*) = 0.80 − 0.045 pH. Therefore, the number of protons (*m*) in the* trans*-resveratrol oxidation was equal to the number of *n*_*a*_ according to the Nernst equation *E*_*p*_ = *E*^0^ − 0.059(*m*/*n*_*a*_) pH. Based on the above results, the* trans*-resveratrol oxidation on the ERGO/GC electrode involved 1 electron and 1 proton, which was consistent with the reported electrochemical oxidation [29, 38, 39].

### 3.4. Quantitative Detection of* trans*-Resveratrol

**Figure 5:**
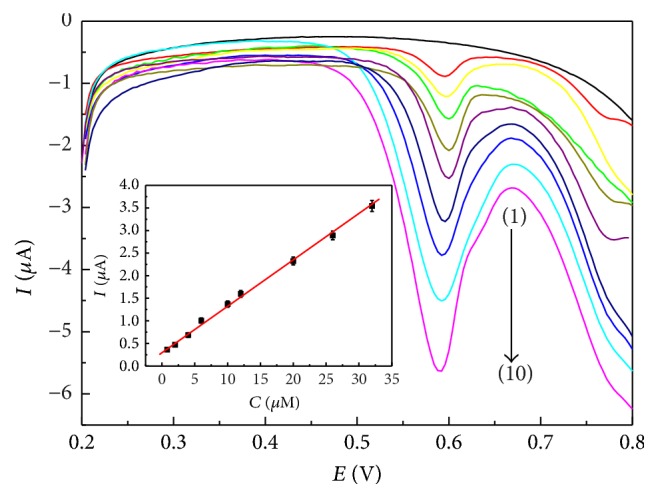
DPV curves at the ERGO/GC electrode for an increasing concentration of* trans*-resveratrol in the 0.04 M BR buffer solution (pH 2.0): (1) 0, (2) 0.8, (3) 2, (4) 4, (5) 6, (6) 10, (7) 12, (8) 20, (9) 26, and (10) 32 *μ*M. Inset shows the calibration curve of oxidation peak current versus concentration.

The sensitivity of the ERGO/GC electrode was evaluated using a typical voltammetric response after the addition of successive aliquots of* trans*-resveratrol to the 0.04 M BR buffer solution (pH 2.0), as shown in Figure 5. The oxidation peak current of* trans*-resveratrol at the ERGO/GC electrode kept on increasing with the increasing concentration of* trans*-resveratrol under identical conditions. In the inset of Figure 5, the calibration of the ERGO/GC electrode to* trans*-resveratrol illustrated that *I*_*p*_ was proportional to the concentration of* trans*-resveratrol. The response to* trans*-resveratrol was linear in the range from 0.8 *μ*M to 32 *μ*M (*R* = 0.998), and a sensitivity of 0.1 *μ*A *μ*M^−1^ could be calculated from the slope of the linear range. The detection limit was 0.2 *μ*M at a signal-to-noise ratio of 3, which was lower than or comparable to those obtained by other electrochemical sensors [23–31], liquid chromatography [5–11], capillary electrophoresis [12–15], fluorescent assays [16, 17], spectroscopy technique [18, 19], and molecularly imprinted polymer [20, 21]. The low detection limit could be according to the electron conduction pathways and high surface area of the graphene film and its strong interaction with* trans*-resveratrol. Furthermore, the detection limit of* trans*-resveratrol also met the requirement for evaluation of the* trans*-resveratrol level in red wine (several mg L^−1^).

The stabilities including repeatability, reproducibility, and long-term stability of the ERGO/GC electrode for the* trans*-resveratrol detection were assessed by the measurement of the response to 20 *μ*M* trans*-resveratrol in 0.04 M BR (pH 2.0) solution. 9 successive modifications of ERGO at the same GC electrode were investigated for repeatability under the same conditions, and the relative standard deviation (RSD) was 6.59%. 5 different ERGO/GC electrodes were studied for the reproducibility, and the RSD was 8.71%. For purpose of evaluation of the long-term stability, the developed modified electrode was stored in 0.04 M BR (pH 2.0) solution at 4°C and it retained 95.6% original voltammetric response even after one month. Consequently, the above results revealed the excellent stability of the modified ERGO film due to its poor insolubility.

### 3.5. Analysis of* trans*-Resveratrol in Red Wine

**Figure 6:**
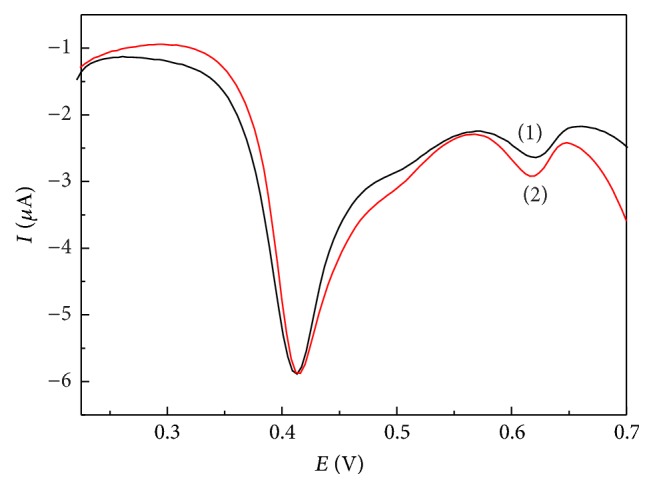
DPV curves of the ERGO/GC electrode before and after the addition of 5 *μ*M* trans*-resveratrol for the red wine sample.

To verify the practicality of the developed method, the red wine residue was diluted using 0.04 M BR (pH 2.0) solution and then was analyzed for the evaluation of the* trans*-resveratrol level at the ERGO/GC electrode. As shown in Figure 6, the oxidation peak of* trans*-resveratrol was observed and a big peak at 0.43 V also appeared owing to other polyphenol substrates in the red wine. The measured* trans*-resveratrol concentration of the wine was 7.90 *μ*M. After the addition of 5 *μ*M* trans*-resveratrol in the wine sample, the recovery was 4.9 *μ*M and the recovery rate was 98%. The above data represented average values of five measurements, and the RSD was 7.3%. Thus, the simplicity, high sensitivity, and anti-interference ability of the ERGO/GC electrode may bring it as the* trans*-resveratrol detector in the food analysis.

## 4. Conclusion

In brief, a simple and highly enhanced sensing platform based on the ERGO film was established for the* trans*-resveratrol detection by DPV. Compared with the reported methods for* trans*-resveratrol detection, the proposed voltammetric sensor provided important advantages as follows. (i) Preparation of the graphene film by one-step electrochemical deposition was a simple, efficient, and green technique. (ii) The sensor was sensitive for* trans*-resveratrol detection due to the strong aromatic *π*-*π* stacking between resveratrol and graphene. (iii) High stability was obtained because of the stability of the graphene coating. (iv) The efficient interaction between* trans*-resveratrol and the graphene film endowed the excellent anti-interference ability in real samples. Therefore, the proposed electrochemical strategy provided valuable information for evaluation of* trans*-resveratrol levels in red wines. The efficient evaluation should get us closer to finding a way for improvement of manufacturing process, the storage control, and the nutrition evaluation of red wines.
